# Impact of prey occupancy and other ecological and anthropogenic factors on tiger distribution in Thailand's western forest complex

**DOI:** 10.1002/ece3.4845

**Published:** 2019-02-18

**Authors:** Somphot Duangchatrasiri, Pornkamol Jornburom, Sitthichai Jinamoy, Anak Pattanvibool, James E. Hines, Todd W. Arnold, John Fieberg, James L. D. Smith

**Affiliations:** ^1^ Wildlife Research Division Department of National Parks, Plant, and Wildlife Conservation Bangkok Thailand; ^2^ University of Minnesota Saint Paul Minnesota; ^3^ Wildlife Conservation Society Thailand Program Nonthaburi Thailand; ^4^ Patuxent Wildlife Research Center U.S. Geological Survey Laurel Maryland

**Keywords:** large landscape survey, multiple scale occupancy, *Panthera tigris*, prey, tiger, Western Forest Complex

## Abstract

1. Despite conservation efforts, large mammals such as tigers (*Panthera tigris*) and their main prey, gaur (*Bos gaurus*), banteng (*Bos javanicus*), and sambar (*Rusa unicolor*), are highly threatened and declining across their entire range. The only large viable source population of tigers in mainland Southeast Asia occurs in Thailand's Western Forest Complex (WEFCOM), an approximately 19,000 km^2^ landscape of 17 contiguous protected areas.
2. We used an occupancy modeling framework, which accounts for imperfect detection, to identify the factors that affect tiger distribution at the approximate scale of a female tiger's home range, 64 km^2^, and site use at a scale of 1‐km^2^. At the larger scale, we estimated the proportion of sites at WEFCOM that were occupied by tigers; at the finer scale, we identified the key variables that influence site‐use and developed a predictive distribution map. At both scales, we examined key anthropogenic and ecological factors that help explain tiger distribution and habitat use, including probabilities of gaur, banteng, and sambar occurrence from a companion study.
3. Occupancy estimated at the 64‐km^2^ scale was primarily influenced by the combined presence of all three large prey species, and 37% or 5,858 km^2 ^of the landscape was predicted to be occupied by tigers. In contrast, site use estimated at the scale of 1 km^2^ was most strongly influenced by the presence of sambar.
4. By modeling occupancy while accounting for imperfect probability of detection, we established reliable benchmark data on the distribution of tigers in WEFCOM. This study also identified factors that limit tiger distributions; which managers can then target to expand tiger distribution and guide recovery elsewhere in Southeast Asia.

## 1 INTRODUCTION

Tigers (*Panthera tigris*) are highly threatened and declining across their entire range. In Thailand, the subspecies, *P.t. corbetti,* has declined from approximately 500 individuals distributed in 13 forest complexes (landscapes) in 1994–1995 (Smith, Tunhikorn, Tanhan, & Simcharoen, and Kanchanasaka, 1999) to an estimated 190–250 in 10 forest complexes in 2004 (Kanchanasaka, Tunhikorn, Vinitpornsawan, Prayoon, & Faengbubpha, 2010). By 2015, a single large viable population remained in the Western Forest Complex (WEFCOM), an approximately 19,000 km^2^ landscape of 17 contiguous protected areas. Three protected areas, Thung Yai East (TYE), Thung Yai West (TYW), and Huai Kha Khaeng (HKK), which together form a World Heritage Site (6,400 km^2^), represent the core of WEFCOM. This core area supports the highest density of tigers on the landscape (Duangchantrasiri et al., 2016; Simcharoen, Pattanavibool, Karanth, Nichols, & Kumar, 2007) and contains one of only four remaining source populations of tigers worldwide that have, under current management, a high probability of being viable for the next 100 years (Kenney, Allendorf, McDougal, & Smith, 2014). Elsewhere in Southeast Asia tiger populations are on the verge of extirpation.

WEFCOM is part of an even larger conservation landscape in the Tenasserim Range, which forms Thailand's western border with Myanmar (WEFCOM, 2004). Because of its size and geographic extent, WEFCOM provides critical habitat for tigers in the region. The landscape also supports numerous other threatened wildlife species including the tiger's main prey: gaur (*Bos gaurus*), banteng (*Bos javanicus*), wild water buffalo (*Bubalus arnee*), and sambar (*Rusa unicolor*) (Simcharoen, Simcharoen, Duangchantrasiria, Bump, & Smith, 2018). Data from camera‐trapping and radio telemetry suggest that HKK alone supports an estimated 35 to 58 tigers (Simcharoen et al., 2007; Duangchantrasiri et al., 2016). Female home ranges there vary from 35 to 105 km^2^ (Simcharoen, Savini, Gale, Simcharoen, et al., 2014). Elsewhere in WEFCOM, tiger distribution and abundance and the quality of habitat are less well known. However, ranger patrols suggest tigers are likely absent from large parts of WEFCOM, and their major prey is similarly reduced (Jornburom, 2016). This lack of knowledge restricts planning efforts to monitor and manage other protected areas in this landscape. To prioritize and strengthen the future protection of tigers throughout WEFCOM, it is important to determine where tigers occur.

Recent studies using occupancy modeling have greatly increased understanding of how habitat connectivity, human disturbance, and prey availability affect tiger occurrence. Important factors impacting tigers include forest type and extent of vegetation cover (Sunarto, Kelly, Parakkasi, & Hutajulu, 2015), connectivity of habitat (Joshi, Vaidyanathan, Mondol, Edgaonkar, & Ramakrishnan, 2013; Yumnam et al., 2014), and anthropogenic impacts such as livestock and human settlements (Harihar & Pandav, 2012; Karanth, Gopalaswamy, Kumar, Nichol, & MacKenzie, 2011; Sunarto et al., 2015). In most of WEFCOM and elsewhere in Thailand, a significant knowledge gap remains about the tiger's distribution. Previous tiger–habitat relationship studies throughout WEFCOM were limited and used inconsistent spatial and temporal scales (Kanchanasaka et al., 2010; Rabinowitz, 1993; Smith, Ahern, & McDougal, 1998; WEFCOM, 2004). They also neglected to account for imperfect detection, which can obscure the underlying ecological processes that determine distribution and habitat relationships, especially when surveys are conducted over a large landscape (Pollock et al., 2002). Therefore, it is difficult to reliably compare results across these studies. To establish reliable benchmark data on tiger distribution patterns, we conducted spatially replicated occupancy surveys throughout WEFCOM, similar to those being applied across other tiger landscapes (Harihar & Pandav, 2012; Hines et al., 2010; Karanth et al., 2011).

Occupancy models applied to multiple spatial scales, with both landscape and fine‐scale predictors, have been demonstrated to be effective in addressing wildlife conservation needs (Scott et al., 2002) and have been used specifically to help inform conservation strategies of multiple species, including tigers (Wikramanayake et al., 2011). At a regional level, landscape‐wide assessment of tiger distribution facilitated the identification of source populations, meta‐population structure, and functional corridors that allow individuals to move through habitat impacted by human disturbances (Karanth et al., 2011; Ranganathan, Chan, Karanth, & Smith, 2008; Smith et al., 1998). At a finer scale, space‐use patterns in source areas provide insights into local factors driving habitat use, which can help inform local management options (Sunarto et al., 2015; Vinitpornsawan, 2013). In summary, analyses that include multiple spatial scales can improve understanding of tiger–habitat relationships (Johnson, 1980; McDonald, Erickson, Boyce, & Alldredge, 2012).

In this study, we seek to: (a) identify how anthropogenic pressures, landscape features, and prey occupancy determine tiger distribution and habitat use, (b) estimate the proportion of area occupied (true occupancy) by tigers at a landscape scale using a 64 km^2^ grid, (c) model habitat relationships of tigers at a 1‐km^2^ scale to determine drivers of habitat use, and (d) develop a predictive distribution map for tigers based on spatially explicit site use models. We conclude by providing guidance on possible alternative management options.

## 2 MATERIALS AND METHODS

### 2.1 Study area

**Figure 1:**
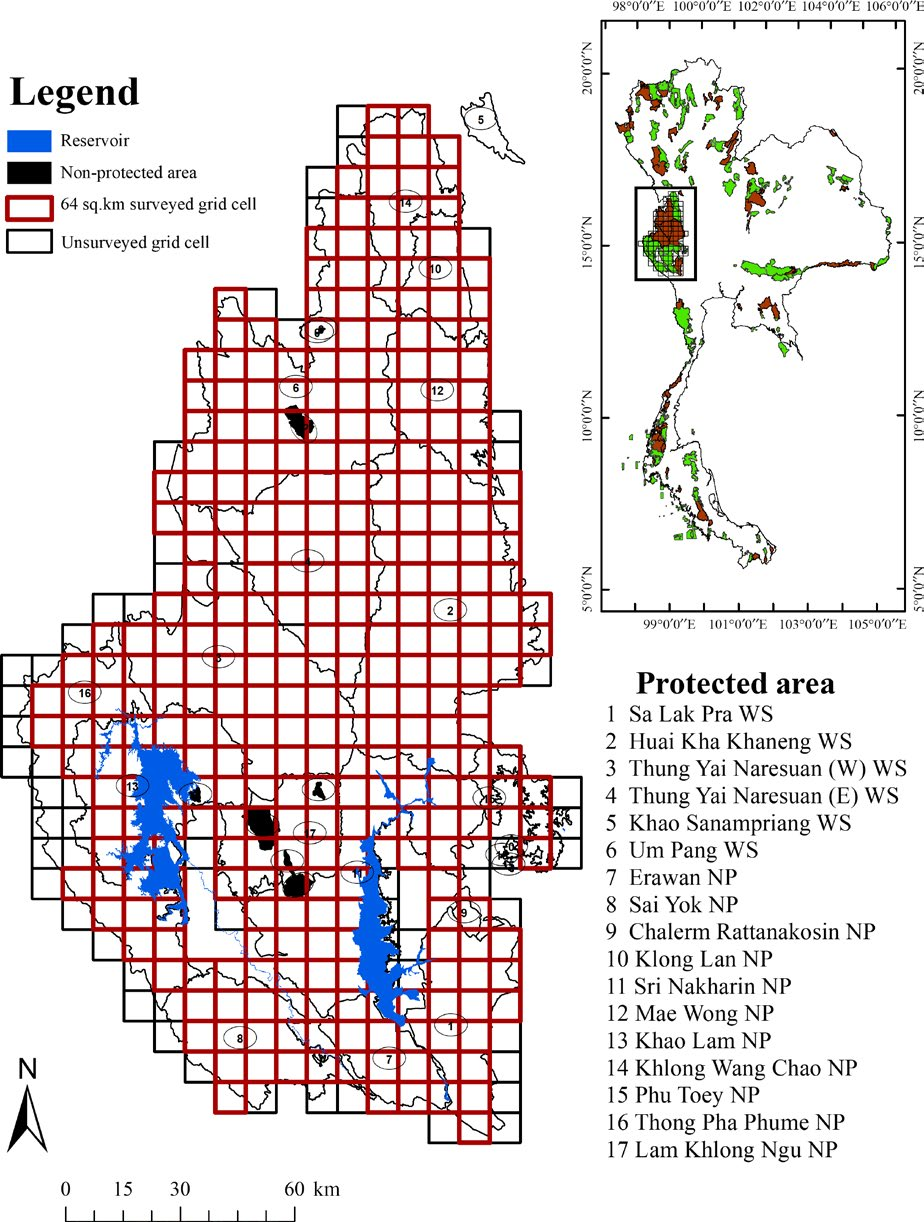
Study area and designed sample units of tiger in Western Forest Complex (WEFCOM), Thailand (2010–2012). The map shows the spatial distribution of surveyed grid cells (those with >10% forest cover). Inset: location of the study area in Thailand is outlined by a red box. NP: National Park; WS: Wildlife Sanctuary.

From November 2010 to December 2012, Thailand's Department of National Parks, Plants, and Wildlife Conservation (DNP), the Wildlife Conservation Society (WCS, Thailand), and the World Wildlife Fund (WWF, Thailand) conducted an occupancy survey that included elephants (*Elephus maximus*), tigers and the main prey of tigers: banteng (*Bos javanicus*), gaur (*Bos gaurus*), and sambar (*Rusa unicolor*) in the Western Forest Complex (WEFCOM) (Figure 1). Collectively, these three large ungulates comprise 88%–95% of the tiger's prey in WEFCOM (Pakpien et al., 2017; Simcharoen et al., 2018). This landscape covers 19,600 km^2 ^and consists of 17 contiguous protected areas making it the largest intact protected area in Thailand and all of southern Asia (Figure 1).

The study area ranges in elevation from 200 to 2,180 m but the dominant elevation varies from 600–1,000 m, and slopes were generally moderate (15%–30%). The major vegetation types include mixed deciduous + bamboo (MD 48.3%), dry evergreen (DE 27.5%), tropical hill evergreen forest (HE 9.6%), savannah grassland (GR 7.3%), agriculture (AG 4.0%), and dry dipterocarp forest (DD 2.2%) (WEFCOM, 2004). Additionally, activities from 139 human communities inside and <3 km from WEFCOM borders greatly affect the complex (WEFCOM, 2004). Evidence indicates these human settlements reduce abundance or led to shifts in distribution of large mammals (Duengkae, Maneerat, Pattanavibool, & Marod, 2004). Despite establishment of 149 ranger stations in WEFCOM, poaching, illegal logging, and harvesting of non‐timber forest products continue to negatively impact the distribution and abundance of tigers and other large mammals (Duangchantrasiri et al., 2016).

### 2.2 Tiger occupancy survey

Our data were collected as part of a multispecies large mammal survey that included elephants, tigers and gaur, banteng, and sambar, the main prey of tigers. We used a 64 km^2^ grid, the approximate size of a female tiger home range, to determine tiger occupancy. Within each grid cell, linear survey routes, composed of 1‐km spatial replicates, were delineated with the number of replicates proportional to the amount of forest habitat within the grid cell. A maximum route length of 15 km per 64‐km^2^ grid cell was used if the entire cell was forested and contained no villages or agricultural land. Grid cells with <10% forest cover were not surveyed under the assumption that they were unlikely to be occupied by tigers. To determine habitat use in addition to occupancy, each 1‐km survey unit was divided into 100‐m spatial replicates. Thus, to estimate occupancy there were a maximum of 15 1‐km replicates and to estimate habitat use within 1‐km^2^ grid cells there were ten 100‐m replicates. Detections (e.g., direct sightings, scats, pugmarks/tracks, carcasses, scent marks, and vocalizations) were recorded in each 100‐m subsegment on typical tiger travel routes that included wildlife trails, mineral licks, forest roads, and river banks. Additionally, in each 100‐m subsegment, substrate condition, habitat type, human activities, and evidence of domestic animals were recorded. Because of the high logistic cost of traveling to random transects, we adopted the widely used modification that reduced travel time by surveying linked replicates along a linear route (Aing, Halls, Oken, Dobrow, & Fieberg, 2011; Hines et al., 2010). Surveys were conducted in the dry season (January–May, October–December) to ensure that scat persistence was consistent. Surveying during the dry season also helps reduce heterogeneity in detection probability induced by rainfall variation (Royle & Nichols, 2003).

This sampling design allowed us to analyze occupancy at two spatial scales or second‐order habitat selection and site use or third‐order habitat selection (Johnson, 1980). For occupancy, we used 64‐km^2 ^grid cells as “sites” and 1‐km segments as spatial replicates. For site use, 1‐km^2 ^grid cells were used as sites and 100‐m segments were used as spatial replicates.

### 2.3 Selection of ecological and anthropogenic variables

We extracted Geographic Information System (GIS)‐based ecological covariates from GIS public domain data and DNP's WEFCOM database. To predict occupancy and site use of tigers, we considered five groups of factors: (a) availability of prey, (b) human disturbance, (c) forest covers (d) distance to streams, and (e) terrain (for sources and further details see Supporting Information Tables S1–S13).

The covariates chosen for modeling were selected based on a priori knowledge of prey and habitat preferences of tigers (Smith et al., 1998; Wegge, Odden, Pokharel, & Storaas, 2009; Harihar & Pandav, 2012). Previous studies in Thailand found that banteng, gaur, and sambar were principal components of tiger diets (Simcharoen et al., 2018). We considered three prey predictors (a) *bovidae*: gaur + banteng, (b) *sambar,* and (c) *all prey* species combined (Jornburom, 2016). Prey covariates were obtained from a companion analysis that estimated probabilities of habitat use (*ψ*) at the site scale of (1‐km^2^), while estimating detection (*p*) at the scale of 0.1 km. All prey was the probability of use by at least 1 of the 3 large prey species. Further, we hypothesized that tigers would be less likely to occur in parts of WEFCOM that experience high human disturbance. We also expected human activity would have a greater impact on occupancy than correlates related to habitat heterogeneity. To assess the impact of human activities, we included a measure of relative abundance of domestic animals (*domestic*), distance from villages (*village*), and distance from roads (road). Forest areas that have been converted to shifting cultivation (*agri*) inside WEFCOM were considered as a measure of habitat degradation.

For habitat‐related covariates, we used Thematic Mapper™ data to calculate the proportion of forest (*forest*), and four separate continuous variables: percentage of hill evergreen forest (*HE*), dry evergreen forest (*DE*), mixed deciduous forest (*MD*), and dry dipterocarp forest (*DD*) (WEFCOM, 2004). For geophysical variables, we used a digital elevation model to obtain elevation, slope, and terrain ruggedness. In addition, distance to rivers and streams (*stream*) and low slope areas (<10% slope) within 1‐km or 3‐km buffers along streams (*flat1km or flat3km*) were also included as covariates that reflect habitat quality near streams (Linkie, Chapron, Martyr, Holden, & Leader‐Williams, 2006). These spatial covariates were calculated using ArcMap 10.3 (ESRI) and ERDAS IMAGINE 2013 software.

Field‐based covariates were collected by surveyors as they walked transects searching for signs of humans and domestic animals including tracks, tree cutting, gun shells, and campfires. Substrate condition (*SUB*), which was hypothesized to impact detection, was recorded as soft soil, hard soil, or leaf litter. The presence of domestic animals (*domestic animal*) was a binary variable coded as “1” for presence or “0” for absence.

### 2.4 Data analyses

We applied a first‐order Markovian model (Hines et al., 2010; Karanth et al., 2011) to account for spatial dependence between adjacent replicates. This model compensates for the lack of spatial independence of replicate surveys by including segment‐level occupancy (parameterized by *θ* ^0^, *θ* ^0^ and *θ^π^*) and detection probability (*p*) conditional on neighboring segment‐level occupancy. The spatial dependence in segment‐level occupancy (*θ*) is captured by *θ* ^0^ if the species is present locally but was not present in the previous spatial replicate, *θ* ^1^ if the species is present locally and was present in the previous spatial replicate, and *θ^π^* for the first replicate where there is no prior information to inform segment‐level occupancy.

We developed models depicting habitat relationships at two spatial scales to better understand tiger ecology and to address conservation needs (Johnson, 1980). We used different subscripts on $\hat{\psi }$ to differentiate estimates at these two scales; ${\hat{\psi }}_{64}$ refers to occupancy probability at the home‐range (64‐km^2^) scale, whereas ${\hat{\psi }}_{1}$ refers to probability of site use at the 1‐km^2^ scale. We modeled probabilities of occupancy (ψ)and detection (*p*) of tigers at both scales as linear functions of the above‐mentioned covariates using a logit link function (MacKenzie et al., 2002).

#### 2.4.1 Model development

To analyze occupancy and site use, we used a sequence of four steps described in more detail below. First, we examined all potential covariates and eliminated highly correlated variables. Next, we grouped ecological correlates into five categories and selected one covariate in each category for subsequent analysis. These variables were additive with no interaction terms. These five variables served as a global occupancy model (Burnham & Anderson, 2002; Duren, Buler, Jones, & Williams, 2011). Third, we held the global model constant to select the most important detection covariate for each scale. Finally, we used the best detection model from step 3 to examine our candidate set of models to identify the most important predictors for occupancy and site use (Karanth et al., 2011; Royle & Nichols, 2003).

To select the appropriate covariates to develop occupancy models, we first explored the correlations among ecological variables to avoid collinearity. We considered correlation coefficients <0.7 as acceptable to include two ecological variables (Dormann et al., 2013). For correlated variables ≥0.7, we selected the covariate considered most representative based on its ecological relevance and availability across a wider area (Fieberg & Johnson, 2015; Giudice, Fieberg, & Lenarz, 2012).

We grouped variables into five categories: availability of prey, human disturbance, forest cover, proximity to stream, and terrain (Table 1). To select the factors included in our “global model,” we modeled covariates in each category in a univariate fashion and chose the factor with the lowest AIC value in each category to incorporate into our global model.

**Table 1:** Model selection results and estimated coefficients (*β*(*SE*)) for best‐supported models of tiger occupancy estimates at 64‐km^2^scale (*ψ*_64_) and 1‐km^2^ scale (*ψ*_1_)

Model^a^ Tiger *ψ*_64_	*ωi* b	*K* c	Dev.^d^	*β*_0_ (*SE*)^e^	Estimated * β *(*SE*)^e^				
					*Prey*	*Forest*	*Elevation*	*Domestic animal*	*Stream*
(All prey + forest + elev + domestic + stream)	0.58	11	1,318.84	−2.08 (0.49)	1.20 (0.37)	1.19 (0.51)	0.72 (0.30)	−0.85 (0.17)	−1.59 (0.32)

Model^a^ Tiger *ψ*_1_	*ωi* b	*K* c	Dev.^d^	*β*_0_ (*SE*)^e^	Estimated *β *(*SE*)^e^		
					*Sambar*	*Stream*	*Domestic animal*
(*Sambar* + *stream* + *domestic*)	0.99	9	3,701.16	−2.24 (0.23)	1.61 (0.05)	−0.54 (0.15)	−3.33 (0.10)

a The model specification for the parameters at 64‐km^2^scale (*ψ*_64_) *θ* ^0^, *θ* ^1^, *θ^π^*, and *p_t_* was: *θ* ^0^(.), *θ* ^1^(.), *p_t_* (flat3km)[<10% slope within 3‐km buffers along streams], *θ^π^*(.) and at 1‐km^2^scale (*ψ*_1_) *θ* ^0^, *θ* ^1^, *θ^π^*, and *p_t_* was: *θ* ^0^(.), *θ* ^1^(.), *p_t_* (flat1km)[<10% slope within 1‐km buffers along streams], *θ^π^*(.).

b The AICc model weight.

c Number of parameters.

d Twice the negative log likelihood.

e Effect sizes (beta estimates) are based on standardized data. See Appendix 1 for a complete list of occupancy models.

We then used the global model for occupancy to evaluate variables that influenced detection. Detection probability (*p*) was modeled as a function of substrate type, presence of domestic animals, and area of low slope (<10% slope) within 1 km or 3 km from streams. In this step, we held occupancy constant using the “global model” (MacKenzie et al., 2006).

The final step was to use the best model for detection to evaluate a set of models to predict occupancy at the 64 km^2^ scale and site use at 1 km^2^ scale (Karanth et al., 2011). For these models, we kept *θ* ^0^, *θ* ^1^, and *θ^π^* constant (Karanth et al., 2011; Thapa & Kelly, 2017). Estimates of coefficients and standard errors (*β*, *SE*(*β*)) were used to determine effect sizes and direction of influence of covariates on the probabilities of occupancy, site use, and detection. Prior to modelling, all continuous covariates were centered and scaled, $\left(x_{i}-{\overset{¯}{x}}_{i}\right)/SD\left(x_{i}\right)$, to facilitate estimation of parameters using numerical optimization techniques (Donovan & Hines, 2007) and to facilitate comparisons among competing variables.

Estimates of regression parameters and the associated variance–covariance matrix derived from program PRESENCE version 12.7 (Hines, 2006) were used to explore the effect of individual covariates while holding all other covariates constant. We calculated variances of predicted values using the delta method as implemented in the CAR package in R (Fox, 2016).

#### 2.4.2 Estimation of overall tiger occupancy

To estimate overall tiger habitat occupancy (${\hat{\psi }}_{64}$) within WEFCOM (total proportion of the landscape occupied by tigers taking into account imperfect detection), we used the top ranked model (Burnham & Anderson, 2002) and averaged predictions from all 309 grid cells. Because our sampling was a near‐complete survey of the WEFCOM landscape with 309 grids of 64 km^2^, we were able to estimate overall tiger occupancy in the WEFCOM as $\hat{\overset{¯}{\Psi }}=\frac{\sum_{i=1}^{309}a_{i}{\hat{\psi }}_{i}}{15,672}$ where *a_i_* is the forested area in cell *i* (i.e., total area of potential tiger habitat is 15,672 km^2^) (Karanth et al., 2011; Srivathsa, Karanth, Jathanna, Kumar, & Karanth, 2014). We used a parametric bootstrap (Efron & Tibshirani, 1994) to compute covariance and the standard error of overall tiger occupancy (${\hat{\psi }}_{64}$).

To map tiger distribution, we employed our best‐supported model for tiger site use (1 km^2^) to help managers identify the key factors impacting spatial distribution of tigers (Lakshminarayanan, Karanth, Goswami, Vaidyanathan, & Karanth, 2015). Mapping at the scale of occupancy (64 km^2^) or use (1 km^2^) produced very similar maps, but the finer‐scale map provides better visualization of habitat use that managers need in making decisions. We used a probability of use >0.6 as a convenient metric to indicate high‐quality tiger habitat.

## 3 RESULTS

We surveyed a total of 3,517 1‐km segments distributed in 309 (64 km^2^) grid cells across WEFCOM to determine occupancy. Further, each segment was subsampled to produce 35,170 100‐m subsegments to evaluate habitat use at a fine scale. We detected tiger sign in 82 of 309 grid cells, which yielded naïve occupancy of 0.27.

Exploratory analysis revealed substantial correlation between distance from villages and all variables estimating prey site use: village and gaur (Pearson's *r* = 0.81), village and sambar (*r* = 0.79), village and Bovidae (*r* = 0.77), and village and all prey (*r* = 0.81). Therefore, we dropped distance from villages from further analysis, but emphasize that the high positive correlation with prey demonstrates that distance from villages was an important driver of prey occupancy (Supporting Information Tables S1–S13).

### 3.1 Influence of covariates on tiger occupancy and site use

The global model at the 64‐km^2 ^scale included the variables: all prey, proportion of forest, elevation, distance from streams, and relative abundance of domestic animals. At the 64‐km^2 ^scale of site use, the same ecological variables were chosen, except “all prey” was replaced by “sambar.” At both scales, we found that the model incorporating low slope areas along streams was the best model for detection.

**Figure 2:**
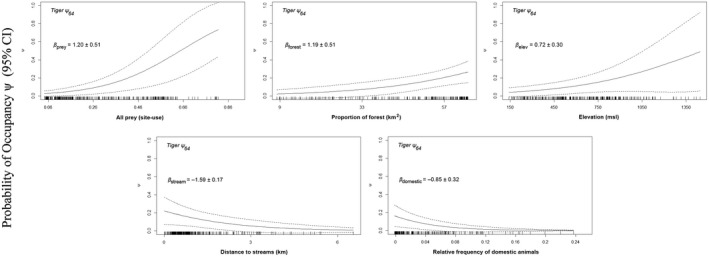
Relationship between the highly influential covariates (based on regression coefficient (*β*) and 95% CI from best‐supported model) and the probability of tiger occupancy in WEFCOM, Thailand (2010–2012). Effect sizes (beta estimates) are based on standardized data while holding the other covariates at their mean values. Tick marks on the *X*‐axis show density of data values in 64 km^2^ grid cell. See Supporting Information Tables 1 and 2 for description of covariates

At the home‐range scale (64‐km^2^), all prey together (i.e., gaur, banteng, and sambar) was the most important predictor of occupancy based on the size of standardized regression coefficients (*β*) (Table 1). Other important factors for predicting occupancy were proportion of forest, elevation, relative abundance of domestic animals, and distance from streams. The AIC‐best model with 58% of the model weight revealed that prey availability, proportion of forest, and elevation were positively correlated with tiger occupancy, and relative abundance of domestic animals was negatively correlated with tiger occupancy (Table 1). The probability of tiger occupancy increased from 20% to 80% as the probability of “all prey” site use increased from approximately 30% to 80% when holding other variables at their mean (Figure 2). A complete set of 32 models and model‐specific regression coefficients are presented in Supporting Information Tables S1–S13.

**Figure 3:**
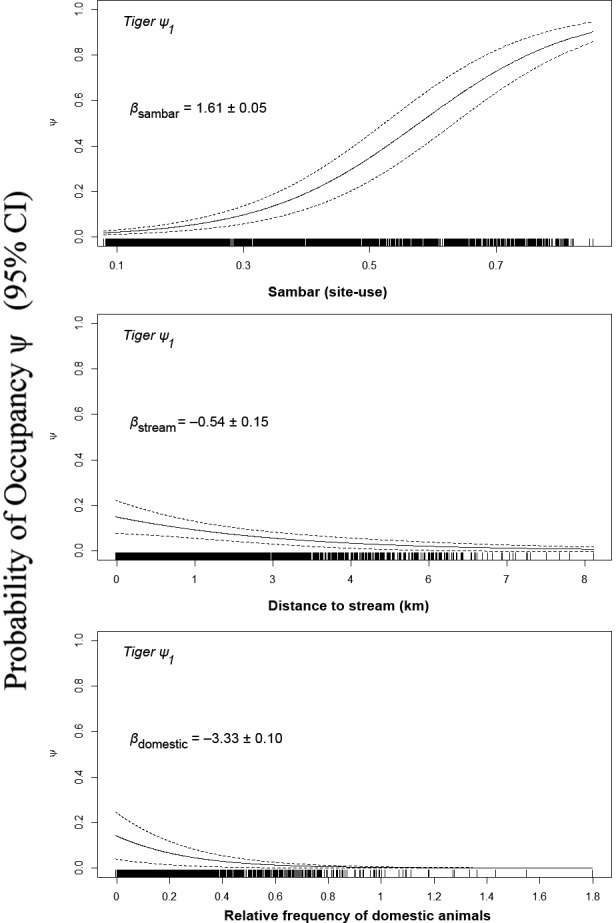
Relationship between the highly influential covariates (based on regression coefficient (*β*) and 95% CI from best‐supported model) and the probability of tiger site use in WEFCOM, Thailand, 2010–2012. Effect sizes (beta estimates) are based on standardized data. Tick marks on the *X*‐axis show density of data values in 1 km^2^ grid cell. See Appendix 1 for description of covariates

For site use (1 km^2^), the most important predictor was sambar presence. In addition to sambar, the best‐supported model included a negative effect of distance from streams and negative effect of domestic livestock. This model garnered 99% of the model weights (Table 1). The probability of tiger site use increased from 20% to 80% as the probability of sambar occupancy increased from approximately 40% to 75% when holding other predictors at their mean values (Figure 3). Model selection results and coefficient estimates are presented in Supporting Information Tables S1–S13.

**Figure 4:**
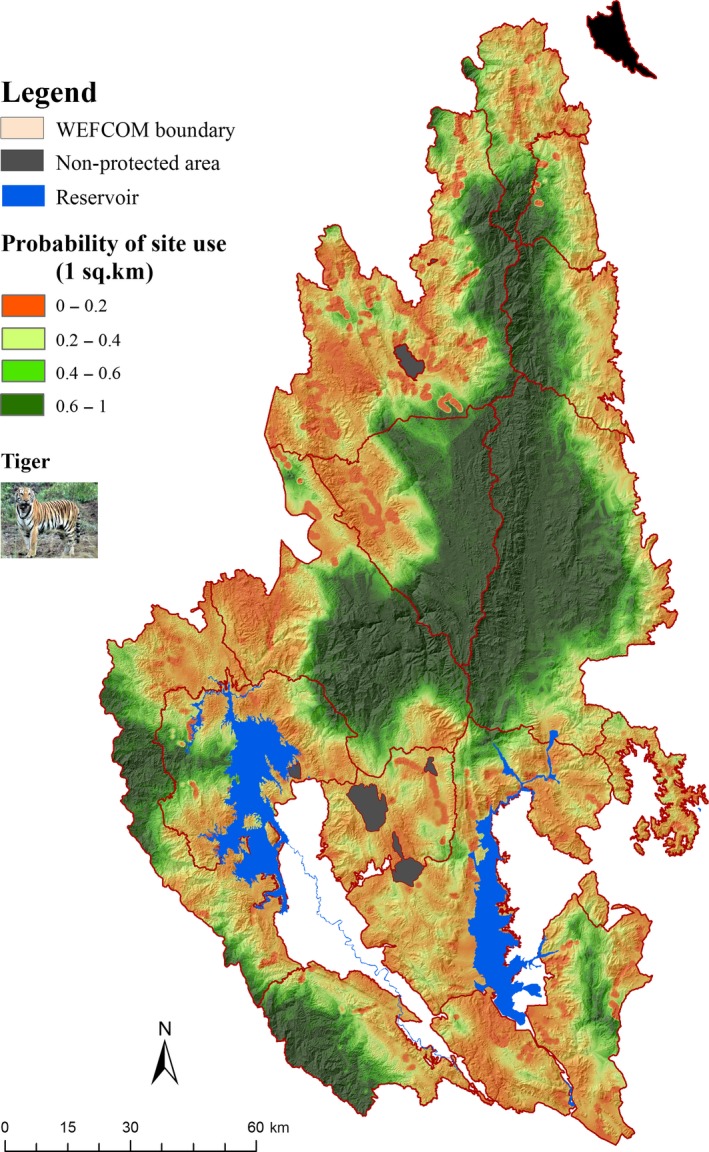
Spatially explicit predictions map of tiger site use constructed from the best‐supported occupancy model developed at 1‐km^2^ scale based on the analysis of occupancy surveys (2010–2012) in WEFCOM, Thailand

We generated a predicted distribution map using model‐based probabilities of tiger site use ≥0.60 (ranging from 0 to 1) from the best‐supported model for site use. Areas of predicted tiger site use (≥0.60) were restricted to the east‐central and northeastern regions of WEFCOM (Huai Kha Khaeng, east and west Thung Yai, Umpang, and Mae Wong) (Figure 4). Site use throughout much of the remainder of the landscape was less contiguous, consisting largely of scattered ‘“islands”' of predicted (site use) ≥0.60.

### 3.2 Estimate of tiger occupancy

We estimated tiger occupancy ${\hat{\psi }}_{64}$ to be 0.37 (*SE* 0.06) for the 309 surveyed cells or 5,858 km^2^ (*SE* 1,758 km^2^) of the 15,600 km^2^ of potential habitat. This estimate was 21% larger than the naïve estimate of 0.27.

## 4 DISCUSSION

Our study is the first modeling effort to incorporate occupancy of prey species as a covariate in tiger occupancy models. Despite widespread use of occupancy modeling, very little research has been published on how prey occupancy influences the distribution of large carnivores (Andresen, Everatt, & Somers, 2014; Everatt, Andresen, & Somers, 2015). Incorporation of prey variables into models has often been done using indices such as overall prey biomass or density (Robinson, Bustos, & Roemer, 2014), general presence or absence of prey (Alexander, Gopalaswamy, Shi, Hughes, & Riordan, 2016; Vinitpornsawan, 2013), relative abundance indices (Chanchani, Noon, Bailey, & Warrier, 2016), or photo‐trap rates (Sunarto et al., 2015). Our study considered only the probability of large prey occupancy because large prey comprises 89% of tiger prey biomass (Simcharoen et al., 2018). We also examined other natural and anthropogenic features to determine the relative influence of these correlates in shaping tiger distribution in WEFCOM. Identification of environmental and anthropogenic factors affecting the distribution of tigers not only increases our understanding of tiger occupancy, but also helps target those correlates that can be managed to increase tiger distribution (Fieberg & Johnson, 2015).

Our study analyzed occupancy at two spatial scales and yielded an important difference. At the 64‐km^2^ scale, the model with all large prey was the highest ranked model. Total large prey biomass was also highly inversely correlated to the size of a female tiger's home range (Simcharoen, Savini, Gale, Simcharoen, et al., 2014). However, within a tiger's home range, tigers preferred localized areas dominated by sambar. This result is consistent with the fact that across the tiger's range, sambar is both a preferred prey and the dominant prey biomass in the diet of tigers (Hayward, Jędrzejewski, & Jedrzejewska, 2012). These differences show the importance of analyzing data at multiple scales.

While tiger occupancy is largely influenced by prey availability, we also found that tiger occupancy decreased with greater distance from streams (Figure 3). We suspect these areas are crucial for tiger site use because low slope forests near streams are also preferred habitat of sambar and banteng (Jornburom, 2016; Simcharoen, Savini, Gale, Roche, et al., 2014). Tigers also occupied areas of higher altitude in the core of WEFCOM; this finding might be attributed to avoiding human activities near villages, especially in the west and the south of WEFCOM. Thus low elevation areas per se may not limit tigers, but, instead, livestock grazing at low elevation may degrade the habitat of the tiger's primary prey. Also, poaching sign is more common at lower elevations (Jornburom, 2016; Vinitpornsawan, 2013).

Our study demonstrated that large parts (63%) of the WEFCOM landscape were devoid of tigers and that tiger habitat use was concentrated in core protected areas in the north (Figure 4), whereas there were only a few scattered patches that were identified as potential habitat in southern areas where tigers were historically widely distributed (Smith et al., 1999). These current patterns of tiger distribution show a positive response to the presence of large ungulates and a negative response to domestic cattle grazing. Previous research has shown the relationship of tiger density to prey density (Karanth, Nichols, Kumar, Link, & Hines, 2004; Simcharoen, Savini, Gale, Simcharoen, et al., 2014). Given that distribution and abundance of large ungulates are critical to the distribution and abundance of tigers, additional research is needed to identify the key ecological correlates that drive ungulate distribution and abundance in western Thailand.

## 5 CONCLUSIONS

Our results suggest that low tiger occurrence in WEFCOM is primarily due to low abundance of large prey and Jornburom (2016) attributed their low abundance to human activities near villages. Several other occupancy studies note that scarcity of natural prey near villages is a consequence of degradation of habitat by livestock grazing (Harihar & Pandav, 2012; Karanth et al., 2011). However, relocating villagers, who have long historical residence, may not be an accepted management strategy. Furthermore, globally there can be strong opposition to forced resettlement (Clements, Suon, Wilkie, & Milner‐Gulland, 2014). The well‐established Smart Patrolling system has had an overall strong protection impact in WEFCOM, but has not increased prey abundance or eliminated domestic livestock grazing or subsistence poaching near villages (Duangchantrasiri et al., 2016). Thus increasing tiger population size may depend on reducing certain activities in the vicinity of villages.

Jornburom (2016) modeled the potential significant increase in tiger prey distribution if such a scenario applied to just nine strategically located villages in Thung Yai East and West. Her model examined the impact on tigers if sufficient incentives and co‐management reduced livestock grazing and subsistence hunting near villages. Currently, a grant from United Nations Development Program's Global Environmental Facility has funded two Thai Non‐governmental Organizations to establish pilot programs that are targeting nine Karen villages embedded in Thung Yai East (seven villages) and Thung Yai West (two villages) with the goal of establishing co‐management (United Nations Development Program, 2015). Specific objectives of co‐management in these and future studies could be beneficial to tiger conservation. In a similar situation in Nepal, co‐management has been effective in reducing poaching and habitat degradation in forests in close proximity to villages (Carter, Shrestha, Karki, Pradhan, & Liu, 2012).

Our research shows that the low occupancy of tigers (37%) was largely a consequence of the absence of large prey. Managers need to identify the factors that limit the distribution of large mammals and test new options to increase large ungulate distribution so that WEFCOM remains source population able to support tiger restoration efforts elsewhere in Thailand and Southeast Asia.

## CONFLICT OF INTEREST

None declared.

## AUTHORS' CONTRIBUTIONS

SD, SJ, PJ, AP conceived the ideas and designed field methodology; SD, PJ, SJ collected the data; PJ, JH, TA, JF, JS analyzed the data; PJ, JF, TA, JS contributed critically to the drafts and all authors gave final approval for publication.

## Data Availability

Presence/Absence of tiger in WEFCOM Thailand: Dryad https://doi.org/10.5061/dryad.7h8f15s.
