# Corrigendum

**DOI:** 10.1002/ece3.5242

**Published:** 2019-05-29

In “Impact of prey occupancy and other ecological and anthropogenic factors on tiger distribution in Thailand's western forest complex,” which was published in issue 5, March 2019, the surnames of the first and fourth authors were incorrect. The correct information is printed below.

- Somphot Duangchatrasiri should be Somphot Duangchantrasiri
- Anak Pattanvibool should be Anak Pattanavibool
