# Supplementary material for: Impact of prey occupancy and other ecological and anthropogenic factors on tiger distribution in Thailand's western forest complex
Source: Ecol Evol. 2019 Feb 18;9(5):2449–58. doi: 10.1002/ece3.4845 (PMC6405490; doi:10.1002/ece3.4845)
Supplement: Supplementary file 1 [file ECE3-9-2449-s001.docx]

Supporting Information

**Table 1.** Descriptions, data sources, and means and ranges of measurements for 64 km^2^ and 1 km^2^ grid cells of the field occupancy survey effort for each protected area in the Western Forest Complex (WEFCOM), Thailand

| **Covariate; data used for analysis** | **Data source** | **Mean (range):**  **64 km^2^-scale use** | **Mean (range):**  **1 km^2^-scale use** | **Data type** | **GIS analysis** |
| --- | --- | --- | --- | --- | --- |
| Domestic animals (domestic; sum) | Occupancy survey | 0-0.2 (km)  mean=0.02 (km) | 0-0.2 (km)  mean=0.02 (km) | Relative frequency of domestic animals observations | We used the kernel density estimator in the Spatial Analyst ArcMap extension to create a density surfaces from the relative frequency of domestic animals with a 1-km search radius and output resolution of 30x30 m. Then performed a z-transformation on these data to enter them in PRESENCE. |
| Distance to villages (village; mean) | DNP,  WEFCOM-GIS database | 1.4-30 (km)  mean=8 (km) | 0.2-40 (km)  mean=11 (km) | GPS waypoints of villages located inside and outside 3-km along the boundary of WEFCOM | We used Euclidean Distance tool to create raster coverage that measured the distance of each 30x30 m pixel to each locations of villages located inside and outside 3-km along the boundary of WEFCOM, then performed a z-transformation on these data to enter them in PRESENCE. |
| Distance to roads (road; mean) | DNP,  WEFCOM-GIS database | 0.2-16 (km)  mean=5 (km) | 0.2-20 (km)  mean=4 (km) | GPS shapefiles of road networks within 10 km from the WEFCOM boundary | We used Euclidean Distance tool to create raster coverage that measured the distance of each 30x30 m pixel to each locations of road networks within 10 km from the WEFCOM boundary, then performed a z-transformation on these data to enter them in PRESENCE. |
| Elevation (elevation; median) | DNP,  WEFCOM-GIS database | 140-2,100 (msl)  mean=582 (msl) | 71-1,600 (msl)  mean=595 (msl) | GIS raster data of digital elevation model | Data were extracted from Aster Global Digital  Elevation Model v002 (ASTG TM) at 30 m resolution, then performed a z-transformation on these data to enter them in PRESENCE. |
| Slope (slope; median ) | DNP,  WEFCOM-GIS database | 0-27  mean=12 | 0-36  mean=114 | GIS raster data of slope | Data were extracted from Aster Global Digital  Elevation Model v002 (ASTG TM) at 30 m resolution, then performed a z-transformation on these data to enter them in PRESENCE. |
| Vector Ruggedness Measure (rugged; mean) | DNP,  WEFCOM-GIS database | 0-0.1  mean=0.01 | 0-0.1  mean=0.01 | GIS raster data of Vector Ruggedness Measure | Data were extracted by calculating the dispersion of vectors orthogonal to the landscape surface at 30x30 m resolution. Ruggedness values in the output raster can range from 0 (no terrain variation) to 1 (complete terrain variation), then performed a z-transformation on these data to enter them in PRESENCE. |
| Shallow patch size of low slope areas along the rivers (flat1km or flat3km; sum) | DNP,  WEFCOM-GIS database | flat1km; 0-33 (km^2^)  mean=9 (km^2^)/  flat3km; 0-50 (km^2^)  mean=15 (km^2^) | flat1km; 0-1 (km^2^)  mean=0.2 (km^2^)/  flat3km; 0-1 (km^2^)  mean=0.3 (km^2^) | GIS raster data of areas low slope (<10% slope) buffer from river in 1 km and 3 km. | We use raster calculator in the Spatial Analyst ArcMap extension to combine raster data of low slope and the patch area of 1 km and 3 km-buffered from rivers. We use Spatial Analyst Tools, Zonal Statistics, *TABULATE AREA* tool to calculate total shallow patch size of low slope areas, then performed a z-transformation on these data to enter them in PRESENCE. |
| Distance to stream (stream; mean) | DNP,  WEFCOM-GIS database | 0.5-60 (km)  mean=2 (km) | 0-8 (km)  mean=1 (km) | GIS shapefiles of stream networks, both permanent and seasonal stream | We used Euclidean Distance tool to create raster coverage that measured the distance of each 30x30 m pixel to nearest water both seasonal and permanent stream lines, then performed a z-transformation on these data to enter them in PRESENCE. |
| Proportion of forest covered (forest; sum) | DNP,  WEFCOM-GIS database | 0-64 (km^2^)  mean=43 (km^2^) | - | GeoTIFF files based on satellite imagery representing forest covered | We use Spatial Analyst Tools, Zonal Statistics, *TABULATE AREA* tool to calculate forest total area of forest covered in each grid cell (both 64 and 1 km^2^), then performed a z-transformation on these data to enter them in PRESENCE. |
| Habitat class  (HE,MD,DD,DE,  agri; sum) | DNP,  WEFCOM-GIS database | HE;0-64 (km^2^)  mean=4 (km^2^)/ MD; 0-64 (km^2^)  mean=21/ DD; 0-20 (km^2^)  mean=2/ DE; 0-62 (km^2^)  mean=13/ agri; 0-28 (km^2^)  mean=2 | HE;0-1 (km^2^)  mean=0.2 (km^2^)/ MD; 0-1 (km^2^)  mean=0.4/ DD; 0-20 (km^2^)  mean=2/ DE; 0-1 (km^2^)  mean=0.9/agri; 0-1 (km^2^)  mean=0.2 | GIS shapefiles derived from Landsat imagery (Lansat5), representing land cover types per the Land Cover Classification System (LCCS) of the UN FAO | We accumulated seven LCCS classifications into five relevant habitat classes, Hill evergreen (HE), mixed-deciduous (MD), dry dipterocarp forest (DD), dry evergreen forest (DE) and degraded habitat as agricultural areas (AGR). We use Spatial Analyst Tools, Zonal Statistics, *TABULATE AREA* tool to calculate the proportion of these vegetation class within each grid cell (both 64 and 1 km^2^), then performed a z-transformation on these data to enter them in PRESENCE. |
| Substrate condition (SUB) | Occupancy survey |  |  | Subjective grading of substrate quality for detecting tracks. | 1: soft soil; 2: hard soil; 3: leave litter |
| Main prey  (allprey; gaur+banteng+sambar), bovidae; gaur+banteng, sambar | Occupancy survey | Gaur; 0-1  mean=0.4/ banteng; 0-1  mean=0.1/ sambar; 0-1  mean=0.5/ | Gaur; 0-1  mean=0.4/ banteng; 0-1  mean=0.1/ sambar; 0-1  mean=0.5/ | Probability of prey site-use | We estimated probability of prey site-use at 1 km^2^-scale using occupancy models (P. Jornburom et al. *in press*), then performed a z-transformation on these data to enter them in PRESENCE. |

**Table 2.** List of factors hypothesized to influence patterns of tiger occupancy (64 km^2^), site-use (1 km^2^) and detection probability in Western Forest Complex (WEFCOM), Thailand.

| **Covariate** | **Description** | **Predicted Effect** | |
| --- | --- | --- | --- |
|  |  | **Occupancy** | **Detection** |
| **Survey-specific covariate** | |  |  |
| SUB | Substrate; 1= Soft soil, 2 = Hard soil, 3 = Leaf litter |  | +/- |
| Domestic | Presence of domestic animals (0,1) | - | - |
| **Site-specific covariate** | |  |  |
| **Availability of prey** | | | |
| *bovid | Probability of Bovidae site use (Bovidae; gaur+banteng) | + |  |
| *sambar | Probability of sambar site use | + |  |
| *allprey | Probability of all preferred prey site use | + |  |
| **Human disturbance** | | | |
| Domestic | Relative frequency of domestic animal | - |  |
| Road | Distance to roads (m.) | +/- |  |
| Village | Distance to villages (m.) | - |  |
| Agriculture | Proportion of forest degradation from agricultural clearing inside protected areas | - |  |

^*^Probability of site use for tiger main prey was developed by P. Jornburom 2016

**Table 2**. **(continued)**

| **Covariate** | **Description** | **Predicted Effect** | |
| --- | --- | --- | --- |
|  |  | **Occupancy** | **Detection** |
| **Proximity to stream** | | | |
| Stream | Distance to stream | + | +/- |
| flat1km  flat3km | Patch area along the river with slope <10% (1-km or 3-km buffered) | +/- | +/- |
| **Terrain** |  |  |  |
| Elev | Elevation | +/- |  |
| Slope | Slope | +/- |  |
| Rugged | Vector Ruggedness Measure | +/- |  |
| Forest covers | Proportion of forest area | + |  |
| DD,HE,MD,DE | Proportion of each forest type (DD; Dry dipterocarp, HE; Hill evergreen, MD; Mixed deciduous, DE; Dry evergreen) | +/- |  |

**Table 3.** Model selection results for Step 1; determining the ‘best’ covariate for “global model” structure of tiger at 64 km^2^ grid-scale of occupancy in WEFCOM, Thailand, 2010-2012.

| **Category** | **Model** | **AIC** | **ΔAIC^a^** | ωi^b^ | **Model**  **Likelihood** | ***K^c^*** | **Dev^d^.** |
| --- | --- | --- | --- | --- | --- | --- | --- |
| **Availability** | $\psi$(prey),$\theta^{0}$(.),$\theta^{1}$(.),$p$ (.) $\theta^{\pi}$(.) | 1363.96 | 0 | 0.76 | 1 | 6 | 1351.96 |
| **of prey** | $\psi$(bovid),$\theta^{0}$(.),$\theta^{1}$(.),$p$(.) $\theta^{\pi}$(.) | 1366.6 | 2.64 | 0.20 | 0.27 | 6 | 1354.6 |
|  | $\psi$(sambar),$\theta^{0}$(.),$\theta^{1}$(.),$p$(.) $\theta^{\pi}$(.) | 1369.75 | 5.79 | 0.04 | 0.05 | 6 | 1357.75 |
|  | $\psi$(.),$\theta^{0}$(.),$\theta^{1}$(.),$p$(.) $\theta^{\pi}$(.) | 1459.43 | 95.47 | 0 | 0 | 5 | 1449.43 |
| **human** | $\psi$(domestic),$\theta^{0}$(.),$\theta^{1}$(.),$p$(.) $\theta^{\pi}$(.) | 1409.68 | 0 | 0.97 | 1 | 6 | 1397.68 |
| **disturbance** | $\psi$(agri),$\theta^{0}$(.),$\theta^{1}$(.),$p$(.) $\theta^{\pi}$(.) | 1416.32 | 6.64 | 0.03 | 0.04 | 6 | 1404.32 |
|  | $\psi$(road),$\theta^{0}$(.),$\theta^{1}$(.),$p$(.) $\theta^{\pi}$(.) | 1447.28 | 37.6 | 0 | 0 | 6 | 1435.28 |
|  | $\psi$(.),$\theta^{0}$(.),$\theta^{1}$(.),$p$(.) $\theta^{\pi}$(.) | 1459.43 | 49.75 | 0 | 0 | 5 | 1449.43 |

**Table 3 continued.**

| **Category** | **Model** | **AIC** | **ΔAIC^a^** | ωi^b^ | **Model**  **Likelihood** | ***K^c^*** | **Dev^d^.** |
| --- | --- | --- | --- | --- | --- | --- | --- |
| **Proximity** | $\psi$(stream),$\theta^{0}$(.),$\theta^{1}$(.),$p$(.) $\theta^{\pi}$(.) | 1457.22 | 0 | 0.58 | 1 | 6 | 1445.22 |
| **to stream** | $\psi$(.),$\theta^{0}$(.),$\theta^{1}$(.),$p$(.) $\theta^{\pi}$(.) | 1459.43 | 2.21 | 0.19 | 0.33 | 5 | 1449.43 |
|  | $\psi$(flat1km),$\theta^{0}$(.),$\theta^{1}$(.),$p$(.) $\theta^{\pi}$(.) | 1459.85 | 2.63 | 0.16 | 0.27 | 6 | 1447.85 |
|  | $\psi$(flat3km),$\theta^{0}$(.),$\theta^{1}$(.),$p$(.) $\theta^{\pi}$(.) | 1461.39 | 4.17 | 0.07 | 0.12 | 6 | 1449.39 |
| **Terrain** | $\psi$(elevation),$\theta^{0}$(.),$\theta^{1}$(.),$p$(.) $\theta^{\pi}$(.) | 1441.92 | 0 | 1 | 1 | 6 | 1429.92 |
|  | $\psi$(slope),$\theta^{0}$(.),$\theta^{1}$(.),$p$(.) $\theta^{\pi}$(.) | 1455.76 | 13.84 | 0 | 0 | 6 | 1443.76 |
|  | $\psi$(rugged),$\theta^{0}$(.),$\theta^{1}$(.),$p$(.) $\theta^{\pi}$(.) | 1457.63 | 15.71 | 0 | 0 | 6 | 1445.63 |
|  | $\psi$(.),$\theta^{0}$(.),$\theta^{1}$(.),$p$(.) $\theta^{\pi}$(.) | 1459.43 | 17.51 | 0 | 0 | 5 | 1449.43 |

**Table 3 continued.**

| **Category** | **Model** | **AIC** | **ΔAIC^a^** | ωi^b^ | **Model**  **Likelihood** | ***K^c^*** | **Dev^d^.** |
| --- | --- | --- | --- | --- | --- | --- | --- |
| **Forest covers** | $\psi$(forest),$\theta^{0}$(.),$\theta^{1}$(.),$p$(.) $\theta^{\pi}$(.) | 1427.13 | 0 | 0.90 | 1 | 6 | 1415.13 |
|  | $\psi$(HE),$\theta^{0}$(.),$\theta^{1}$(.),$p$(.) $\theta^{\pi}$(.) | 1431.46 | 4.33 | 0.10 | 0.11 | 6 | 1419.46 |
|  | $\psi$(DD),$\theta^{0}$(.),$\theta^{1}$(.),$p$(.) $\theta^{\pi}$(.) | 1452.57 | 25.44 | 0 | 0 | 6 | 1440.57 |
|  | $\psi$(DE),$\theta^{0}$(.),$\theta^{1}$(.),$p$(.) $\theta^{\pi}$(.) | 1453.94 | 26.81 | 0 | 0 | 6 | 1441.94 |
|  | $\psi$(MD),$\theta^{0}$(.),$\theta^{1}$(.),$p$(.) $\theta^{\pi}$(.) | 1456.26 | 29.13 | 0 | 0 | 6 | 1444.26 |
|  | $\psi$(.),$\theta^{0}$(.),$\theta^{1}$(.),$p$(.) $\theta^{\pi}$(.) | 1459.43 | 32.3 | 0 | 0 | 5 | 1449.43 |

*^a^Difference in adjusted Akaike information criterion (AIC) score between the best-supported model and any given model. ^b^The AICc model weight, ^c^Number of parameters, ^d^Twice the negative log likelihood. See* ***Appendix 1*** *for a complete list of occupancy models.*

**Table 4.** Model selection results for Step 1; determining the ‘best’ covariate for “global model” structure of tiger at 1 km^2^ grid-scale of occupancy in WEFCOM, Thailand, 2010-2012.

| **Category** | **Model** | **AIC** | **ΔAIC^a^** | ωi^b^ | **Model**  **Likelihood** | ***K^c^*** | **Dev^d^.** |
| --- | --- | --- | --- | --- | --- | --- | --- |
| **Availability** | $\Psi$(sambar),$\theta^{0}$(.),$\theta^{1}$(.),$p$(.) $\theta^{\pi}$(.) | 3949.61 | 0 | 0.33 | 1 | 6 | 3937.61 |
| **of prey** | $\psi$(prey),$\theta^{0}$(.),$\theta^{1}$(.),$p$(.) $\theta^{\pi}$(.) | 3950.26 | 0.65 | 0.24 | 0.72 | 6 | 3938.26 |
|  | $\psi$(bovid),$\theta^{0}$(.),$\theta^{1}$(.),$p$(.) $\theta^{\pi}$(.) | 3950.27 | 0.66 | 0.24 | 0.72 | 6 | 3938.27 |
|  | $\psi$(.),$\theta^{0}$(.),$\theta^{1}$(.),$p$(.) $\theta^{\pi}$(.) | 3953.32 | 3.71 | 0.05 | 0.16 | 5 | 3943.32 |
| **Human** | $\psi$(domestic),$\theta^{0}$(.),$\theta^{1}$(.),$p$(.) $\theta^{\pi}$(.) | 3943.22 | 0 | 0.97 | 1 | 6 | 3931.22 |
| **disturbance** | $\psi$(road),$\theta^{0}$(.),$\theta^{1}$(.),$p$(.) $\theta^{\pi}$(.) | 3950.62 | 7.4 | 0.02 | 0.02 | 6 | 3938.62 |
|  | $\psi$(.),$\theta^{0}$(.),$\theta^{1}$(.),$p$(.) $\theta^{\pi}$(.) | 3953.32 | 10.1 | 0.01 | 0.01 | 5 | 3943.32 |
|  | $\psi$(agri),$\theta^{0}$(.),$\theta^{1}$(.),$p$(.) $\theta^{\pi}$(.) | 3954.68 | 11.46 | 0.00 | 0.00 | 6 | 3942.68 |

*.*

**Table 4. continued.**

| **Category** | **Model** | **AIC** | **ΔAIC^a^** | ωi^b^ | **Model**  **Likelihood** | ***K^c^*** | **Dev^d^.** |
| --- | --- | --- | --- | --- | --- | --- | --- |
| **Proximity** | $\psi$(stream),$\theta^{0}$(.),$\theta^{1}$(.),$p$(.) $\theta^{\pi}$(.) | 3947.88 | 0 | 0.85 | 1 | 6 | 3935.88 |
| **to streams** | $\psi$(flat1km),$\theta^{0}$(.),$\theta^{1}$(.),$p$(.) $\theta^{\pi}$(.) | 3953.01 | 5.13 | 0.07 | 0.08 | 6 | 3941.01 |
|  | $\psi$(.),$\theta^{0}$(.),$\theta^{1}$(.),$p$(.) $\theta^{\pi}$(.) | 3953.32 | 5.44 | 0.06 | 0.07 | 5 | 3943.32 |
|  | $\psi$(flat3km),$\theta^{0}$(.),$\theta^{1}$(.),$p$(.) $\theta^{\pi}$(.) | 3954.48 | 6.6 | 0.03 | 0.04 | 6 | 3942.48 |
| **Terrain** | $\psi$(elev),$\theta^{0}$(.),$\theta^{1}$(.),$p$(.) $\theta^{\pi}$(.) | 3950.19 | 0 | 0.76 | 1 | 6 | 3938.19 |
|  | $\psi$(.),$\theta^{0}$(.),$\theta^{1}$(.),$p$(.) $\theta^{\pi}$(.) | 3953.32 | 3.13 | 0.16 | 0.21 | 5 | 3943.32 |
|  | $\psi$(rugged),$\theta^{0}$(.),$\theta^{1}$(.),$p$(.) $\theta^{\pi}$(.) | 3954.82 | 4.63 | 0.08 | 0.10 | 6 | 3942.82 |
|  | $\psi$(slope),$\theta^{0}$(.),$\theta^{1}$(.),$p$(.) $\theta^{\pi}$(.) | 4006.87 | 56.68 | 0 | 0 | 6 | 3994.87 |

**Table 4. continued.**

| **Category** | **Model** | **AIC** | **ΔAIC^a^** | ωi^b^ | **Model**  **Likelihood** | ***K^c^*** | **Dev^d^.** |
| --- | --- | --- | --- | --- | --- | --- | --- |
| **Forest covers** | $\psi$(.),$\theta^{0}$(.),$\theta^{1}$(.),$p$(.) $\theta^{\pi}$(.) | 3953.32 | 0 | 0.36 | 1 | 5 | 3943.32 |
|  | $\psi$(DD),$\theta^{0}$(.),$\theta^{1}$(.),$p$(.) $\theta^{\pi}$(.) | 3954.6 | 1.28 | 0.19 | 0.53 | 6 | 3942.6 |
|  | $\psi$(DE),$\theta^{0}$(.),$\theta^{1}$(.),$p$(.) $\theta^{\pi}$(.) | 3954.97 | 1.65 | 0.16 | 0.44 | 6 | 3942.97 |
|  | $\psi$(HE),$\theta^{0}$(.),$\theta^{1}$(.),$p$(.) $\theta^{\pi}$(.) | 3954.98 | 1.66 | 0.16 | 0.44 | 6 | 3942.98 |
|  | $\psi$(MD),$\theta^{0}$(.),$\theta^{1}$(.),$p$(.) $\theta^{\pi}$(.) | 3955.1 | 1.78 | 0.15 | 0.41 | 6 | 3943.1 |

*^a^Difference in adjusted Akaike information criterion (AIC) score between the best-supported model and any given model. ^b^The AICc model weight, ^c^Number of parameters, ^d^Twice the negative log likelihood. See* ***Supporting Information 1*** *for a complete list of occupancy models.*

**Table 5.** Results of model selection for estimating detection probability ($p$) of tiger at 64 km^2^ grid-scale using 1-km spatial replicates under the constant global model of occupancy in WEFCOM, Thailand, 2010-2012.

| **Model** | **AIC** | **ΔAIC^b^** | ωi^c^ | **Model**  **Likelihood** | ***K^d^*** | **Dev.^e^** |
| --- | --- | --- | --- | --- | --- | --- |
| $\psi$(Global)^a^,$\theta^{0}$(.),$\theta^{1}$(.),$p$(flat3km) $\theta^{\pi}$(.) | 1340.84 | 0 | 0.60 | 1 | 11 | 1318.84 |
| $\psi$(Global),$\theta^{0}$(.),$\theta^{1}$(.),$p$(flat1km) $\theta^{\pi}$(.) | 1341.75 | 0.91 | 0.37 | 0.63 | 11 | 1319.75 |
| $\psi$(Global),$\theta^{0}$(.),$\theta^{1}$(.),$p$(domestic) $\theta^{\pi}$(.) | 1346.86 | 6.02 | 0.03 | 0.05 | 11 | 1324.86 |
| $\psi$(Global),$\theta^{0}$(.),$\theta^{1}$(.),$p$(.) $\theta^{\pi}$(.) | 1351.35 | 10.51 | 0 | 0 | 10 | 1331.35 |
| $\psi$(Global),$\theta^{0}$(.),$\theta^{1}$(.),$p$(SUB) $\theta^{\pi}$(.) | 1358.25 | 17.41 | 0 | 0 | 11 | 1336.25 |
| $\psi$(.),$\theta^{0}$(.),$\theta^{1}$(.),$p$(.) $\theta^{\pi}$(.) | 1459.43 | 118.59 | 0 | 0 | 5 | 1449.43 |

*^a^Constant global model for* ${Tiger}_{\psi_{64}}$*[(prey+forest +elve+stream+domestic)]. ^b^Difference in adjusted Akaike information criterion (AIC) score between the best-supported model and any given model. ^c^The AICc model weight, ^d^Number of parameters, ^e^Twice the negative log likelihood. See* ***Appendix 1*** *for description of covariates*.

**Table 6.** Results of model selection for estimating detection probability ($p$) of tiger at 1-km^2^-scale using 100-m spatial replicates under the constant global model of occupancy in WEFCOM, Thailand, 2010-2012.

| **Model** | **AIC** | **ΔAIC^b^** | ωi^c^ | **Model**  **Likelihood** | ***K^d^*** | **Dev.^e^** |
| --- | --- | --- | --- | --- | --- | --- |
| $\psi$(Global)^a^,$\theta^{0}$(.),$\theta^{1}$(.),$p$(flat1km) $\theta^{\pi}$(.) | 3721.5 | 0 | 0.67 | 1.00 | 11 | 3699.5 |
| $\psi$(Global),$\theta^{0}$(.),$\theta^{1}$(.),$p$(flat3km) $\theta^{\pi}$(.) | 3724.06 | 2.56 | 0.18 | 0.28 | 11 | 3702.06 |
| $\psi$(Global),$\theta^{0}$(.),$\theta^{1}$(.),$p$(domestic) $\theta^{\pi}$(.) | 3725.77 | 4.27 | 0.08 | 0.12 | 11 | 3703.77 |
| $\psi$(Global),$\theta^{0}$(.),$\theta^{1}$(.),$p$(.) $\theta^{\pi}$(.) | 3727.07 | 5.57 | 0.04 | 0.06 | 10 | 3707.07 |
| $\psi$(Global),$\theta^{0}$(.),$\theta^{1}$(.),$p$(SUB) $\theta^{\pi}$(.) | 3727.68 | 6.18 | 0.03 | 0.05 | 11 | 3705.68 |
| $\psi$(.),$\theta^{0}$(.),$\theta^{1}$(.),$p$(.) $\theta^{\pi}$(.) | 3953.32 | 231.82 | 0 | 0 | 5 | 3943.32 |

*^a^Constant global model for* ${Tiger}_{\psi_{1}}$*[(sambar+stream+domestic)]. ^b^Difference in adjusted Akaike information criterion (AIC) score between the best-supported model and any given model. ^c^The AICc model weight, ^d^Number of parameters, ^e^Twice the negative log likelihood. See* ***Appendix 1*** *for description of covariates*.

**Table 7.** Results of model selection to identify ecological and anthropogenic covariates influencing probability of occupancy of ${Tiger}_{\psi_{64}}$ at grid-scale (64 km^2^) (WEFCOM, Thailand, 2010-2012)

| **Model^a^** | **AIC** | **ΔAIC** | ωi | **Model**  **Likelihood** | ***K*** | **Dev.** |
| --- | --- | --- | --- | --- | --- | --- |
| $\psi_{64}$(allprey+forest+elev+stream+domestic) | 1340.84 | 0 | 0.58 | 1 | 11 | 1318.84 |
| $\psi_{64}$(allprey+forest+ elev+domestic) | 1343.35 | 2.51 | 0.16 | 0.28 | 10 | 1323.35 |
| $\psi_{64}$(allprey+elev+stream+domestic) | 1345.02 | 4.18 | 0.07 | 0.12 | 10 | 1325.02 |
| $\psi_{64}$(allprey+forest+ domestic) | 1345.36 | 4.52 | 0.06 | 0.10 | 9 | 1327.36 |
| $\psi_{64}$(allprey+forest+stream+domestic) | 1345.47 | 4.63 | 0.06 | 0.09 | 10 | 1325.47 |
| $\psi_{64}$(allprey+elev+domestic) | 1347.38 | 6.54 | 0.02 | 0.03 | 9 | 1329.38 |
| $\psi_{64}$(allprey+forest+elev+stream) | 1348 | 7.16 | 0.01 | 0.02 | 10 | 1328 |
| $\psi_{64}$(allprey+forest+stream) | 1349.56 | 8.72 | 0 | 0.01 | 9 | 1331.56 |
| $\psi_{64}$(allprey+forest) | 1349.87 | 9.03 | 0 | 0.01 | 8 | 1333.87 |

**Table 7.** **continued.**

| **Model^a^** | **AIC** | **ΔAIC^b^** | $\omega$i^c^ | **Model**  **Likelihood** | ***K^d^*** | **Dev.^e^** |
| --- | --- | --- | --- | --- | --- | --- |
| $\psi_{64}$(allprey+forest+elev) | 1349.99 | 9.15 | 0 | 0.01 | 9 | 1331.99 |
| $\psi_{64}$(allprey+domestic) | 1354.04 | 13.2 | 0 | 0 | 8 | 1338.04 |
| $\psi_{64}$(allprey+elev+stream) | 1354.28 | 13.44 | 0 | 0 | 9 | 1336.28 |
| $\psi_{64}$(allprey+domestic+stream) | 1355.12 | 14.28 | 0 | 0 | 9 | 1337.12 |
| $\psi_{64}$(allprey+elev) | 1355.77 | 14.93 | 0 | 0 | 8 | 1339.77 |
| $\psi_{64}$(allprey) | 1358.89 | 18.05 | 0 | 0 | 7 | 1344.89 |
| $\psi_{64}$(allprey+stream) | 1359.67 | 18.83 | 0 | 0 | 8 | 1343.67 |
| $\psi_{64}$(forest+elev+stream+domestic) | 1367.65 | 26.81 | 0 | 0 | 10 | 1347.65 |
| $\psi_{64}$(forest+elev+domestic) | 1374.42 | 33.58 | 0 | 0 | 9 | 1356.42 |
| $\psi_{64}$(elev+stream+domestic) | 1375.59 | 34.75 | 0 | 0 | 9 | 1357.59 |

**Table 8.** **continued.**

| **Model^a^** | **AIC** | **ΔAIC^b^** | $\omega$i^c^ | **Model**  **Likelihood** | ***K^d^*** | **Dev.^e^** |
| --- | --- | --- | --- | --- | --- | --- |
| $\psi_{64}$(elev+domestic) | 1383.73 | 42.89 | 0 | 0 | 8 | 1367.73 |
| $\psi_{64}$(forest+domestic+stream) | 1383.93 | 43.09 | 0 | 0 | 9 | 1365.93 |
| $\psi_{64}$(forest+domestic) | 1384.2 | 43.36 | 0 | 0 | 8 | 1368.2 |
| $\psi_{64}$(forest+elev+stream) | 1404.11 | 63.27 | 0 | 0 | 9 | 1386.11 |
| $\psi_{64}$(domestic) | 1407.02 | 66.18 | 0 | 0 | 7 | 1393.02 |
| $\psi_{64}$(domestic+stream) | 1407.96 | 67.12 | 0 | 0 | 8 | 1391.96 |
| $\psi_{64}$(forest+elev) | 1412.93 | 72.09 | 0 | 0 | 8 | 1396.93 |
| $\psi_{64}$(forest+stream) | 1415.7 | 74.86 | 0 | 0 | 8 | 1399.7 |
| $\psi_{64}$(forest) | 1419 | 78.16 | 0 | 0 | 7 | 1405 |
| $\psi_{64}$(elev+stream) | 1420.65 | 79.81 | 0 | 0 | 8 | 1404.65 |

**Table 8. countinued.**

| **Model^a^** | **AIC** | **ΔAIC^b^** | $\omega$i^c^ | **Model**  **Likelihood** | ***K^d^*** | **Dev.^e^** |
| --- | --- | --- | --- | --- | --- | --- |
| $\psi_{64}$(elev) | 1430.5 | 89.66 | 0 | 0 | 7 | 1416.5 |
| $\psi_{64}$(stream) | 1449.9 | 109.06 | 0 | 0 | 7 | 1435.9 |
| $\psi_{64}$(),$\theta^{0}$(.),$\theta^{1}$(.),$p(),\theta^{\pi}$(.) | 1459.43 | 118.59 | 0 | 0 | 5 | 1449.43 |

*^a^The model specification for the parameters at 64 km^2^-scale (*$\psi_{64}$*)* $\theta^{0}$*,* $\theta^{1}$*,* $\theta^{\pi}$*, and* $p$ *was:* $\theta^{0}$*(.),*$\theta^{1}$*(.),* $p$*(FLT3km),* $\theta^{\pi}$*(.).^b^Difference in adjusted Akaike information criterion (AIC) score between the best-supported model and any given model. ^c^The AICc model weight, ^d^Number of parameters, ^e^Twice the negative log likelihood. See* **Appendix *1*** *for a complete list of occupancy models.*

**Table 9.** Results of model selection to identify ecological and anthropogenic covariates influencing probability of site-use of ${Tiger}_{\psi_{1}}$ at 1 km^2^-scale (WEFCOM, Thailand, 2010-2012)

| **Model^a^** | **AIC** | **ΔAIC^b^** | $\omega$i^c^ | **Model**  **Likelihood** | ***K^d^*** | **Dev.^e^** |
| --- | --- | --- | --- | --- | --- | --- |
| $\psi_{1}$(sambar+domestic+stream) | 3719.16 | 0 | 0.99 | 1 | 9 | 3701.16 |
| $\psi_{1}$(sambar+domestic) | 3728.33 | 9.17 | 0.01 | 0.01 | 8 | 3712.33 |
| $\psi_{1}$(sambar+stream) | 3922.56 | 203.4 | 0 | 0 | 8 | 3906.56 |
| $\psi_{1}$(sambar) | 3937.05 | 217.89 | 0 | 0 | 7 | 3923.05 |
| $\psi_{1}$(stream) | 3948.06 | 228.9 | 0 | 0 | 7 | 3934.06 |
| $\psi_{1}$(domestic) | 3948.85 | 229.69 | 0 | 0 | 7 | 3934.85 |
| $\psi_{1}$(domestic+stream) | 3950.79 | 231.63 | 0 | 0 | 8 | 3934.79 |
| $\psi_{1}$(),$\theta^{0}$(.),$\theta^{1}$(.),$p(),\theta^{\pi}$(.) | 3953.32 | 234.16 | 0 | 0 | 5 | 3943.32 |

*^a^The model specification for the parameters at 1 km^2^-scale (*$\psi_{1}$*)* $\theta^{0}$*,* $\theta^{1}$*,* $\theta^{\pi}$*, and* $p$ *was:* $\theta^{0}$*(.),*$\theta^{1}$*(.),* $p$*FLT1km),* $\theta^{\pi}$*(.).^b^Difference in adjusted Akaike information criterion (AIC) score between the best-supported model and any given model. ^c^The AICc model weight, ^d^Number of parameters, ^e^Twice the negative log likelihood. See* **Appendix 1** *for a complete list of occupancy models.*

**Table 10** Estimates of untransformed $\beta$ coefficient values (standard errors, SE) of tiger probability of occupancy for the logit link function for different individual covairates based on the models summed AIC weight =1 at grid-scale $\psi_{64}$ in WEFCOM, Thailand

| Model | $\beta_{0}(SE)$ | $\beta_{prey}(SE)$ | $\beta_{forest}(SE)$ | $\beta_{elev}(SE)$ | $\beta_{domestic}(SE)$ | $\beta_{stream}(SE)$ |
| --- | --- | --- | --- | --- | --- | --- |
| $\psi_{64}$(allprey+forest+elev+stream+domestic) | -0.28 (0.43) | 1.20 (0.27)* | 1.20 (0.51)* | 0.72 (0.30)* | -0.85 (0.34)* | -1.59 (0.77)* |
| $\psi_{64}$(allprey+forest+ elev+domestic) | -1.75 (0.39) | 1.22 (0.26)* | 1.18 (0.50)* | 0.50 (0.25) | -0.78 (0.30)* |  |
| $\psi_{64}$(allprey+elev+stream+domestic) | -1.37 (0.28) | 1.24 (0.27)* |  | 0.90 (0.29)* | -0.96 (0.35)* | -1.58 (0.78)* |
| $\psi_{64}$(allprey+forest+ domestic) | -1.85 (0.39) | 1.37 (0.28)* | 1.51 (0.50)* |  | -0.65 (0.30)* |  |
| $\psi_{64}$(allprey+forest+stream+domestic) | -2.06 (0.44) | 1.42 (0.29)* | 1.62 (0.53)* |  | -0.64 (0.31)* | -0.91 (0.68) |
| $\psi_{64}$(allprey+elev+domestic) | -1.03 (0.23) | 1.29 (0.26)* |  | 0.68 (0.25) | -0.87 (0.33) |  |
| $\psi_{64}$(allprey+forest+elev+stream) | -2.14 (0.44) | 1.54 (0.28)* | 1.41 (0.53)* | 0.50 (0.27) |  | -1.41 (0.74) |

*Note: * indicate statistical significance as defined by* $\hat{\beta}\pm1.96 x SE$ *not overlapping 0. Effect sizes (beta estimates) are based on standardized data.*

**Table 11** Estimates of untransformed $\beta$ coefficient values (standard errors, SE) of tiger probability of site-use for for the logit link function for different individual covairates based on the models summed AIC weight =1 at grid-scale $\psi_{1}$ in WEFCOM, Thailand, 2010 -2012.

| Model | $\beta_{0}(SE)$ | $\beta_{sambar}(SE)$ | $\beta_{domestic}(SE)$ | $\beta_{stream}(SE)$ |
| --- | --- | --- | --- | --- |
| $\psi_{1}$(sambar+domestic+stream) | -2.24 (0.23) | 1.61 (0.05)* | -3.33 (0.10)* | -0.54 (0.15)* |
| $\psi_{1}$( sambar+domestic ) | -2.20 (0.23) | 1.14 (0.05)* | -3.10 (0.10)* |  |

*Note: * indicate statistical significance as defined by* $\hat{\beta}\pm1.96 x SE$ *not overlapping 0. Effect sizes (beta estimates) are based on standardized data.*

**Table 12.** Spearman correlation coefficients between the predictor variables at grid-scale (64 km^2^). Those with very high correlations (|r_s_| ≥ 0.60) were not used together in the same model. Number of sites (grid cells) surveyed= 309 of 64 km^2^

|  | sambar | bovidae | allprey | domestic | | road | | village | | agri | | flat1km | | flat3km | | stream | | DD | | MD | | DE | | HE | | forest | | elev | | slope | | rugged | |
| --- | --- | --- | --- | --- | --- | --- | --- | --- | --- | --- | --- | --- | --- | --- | --- | --- | --- | --- | --- | --- | --- | --- | --- | --- | --- | --- | --- | --- | --- | --- | --- | --- | --- |
| sambar | *** | **0.86** | **0.97** | -0.41 | 0.30 | | **0.79** | | -0.46 | | 0.04 | | -0.08 | | -0.14 | | 0.24 | | -0.04 | | 0.17 | | 0.23 | | 0.31 | | 0.20 | | 0.14 | | 0.04 | |  |
| bovidae |  | ***** | **0.96** | -0.31 | 0.24 | | **0.77** | | -0.36 | | 0.02 | | -0.08 | | -0.10 | | 0.26 | | -0.22 | | 0.24 | | 0.37 | | 0.31 | | 0.38 | | 0.14 | | -0.02 | |  |
| allprey |  |  | ***** | -0.37 | | 0.28 | | **0.81** | | -0.43 | | 0.03 | | -0.08 | | -0.13 | | 0.26 | | -0.13 | | 0.21 | | 0.31 | | 0.32 | | 0.30 | | 0.15 | | 0.01 | |
| domestic |  |  |  | ***** | | -0.09 | | -0.35 | | 0.13 | | -0.05 | | 0.01 | | 0.13 | | -0.11 | | -0.04 | | -0.03 | | -0.05 | | -0.01 | | 0.07 | | -0.05 | | 0.02 | |
| road |  |  |  |  | | ***** | | 0.44 | | -0.33 | | -0.07 | | -0.12 | | 0.06 | | 0.02 | | 0.00 | | 0.23 | | 0.19 | | 0.34 | | 0.13 | | 0.33 | | 0.17 | |
| village |  |  |  |  | |  | | ***** | | -0.40 | | 0.09 | | 0.03 | | -0.04 | | 0.21 | | -0.10 | | 0.30 | | 0.31 | | 0.46 | | 0.35 | | 0.27 | | 0.04 | |
| agri |  |  |  |  | |  | |  | | ***** | | 0.13 | | 0.19 | | -0.06 | | -0.15 | | 0.01 | | -0.20 | | -0.11 | | -0.32 | | -0.22 | | -0.17 | | -0.11 | |
| flat1km |  |  |  |  | |  | |  | |  | | ***** | | **0.91** | | -0.45 | | 0.41 | | 0.32 | | -0.28 | | -0.22 | | 0.06 | | -0.44 | | -0.36 | | -0.49 | |
| flat3km |  |  |  |  | |  | |  | |  | |  | | ***** | | -0.32 | | 0.29 | | 0.26 | | -0.18 | | -0.29 | | 0.07 | | -0.40 | | -0.42 | | -0.58 | |
| stream |  |  |  |  | |  | |  | |  | |  | |  | | ***** | | -0.16 | | -0.19 | | 0.33 | | -0.08 | | 0.05 | | 0.22 | | 0.00 | | 0.00 | |
| DD |  |  |  |  | |  | |  | |  | |  | |  | |  | | ***** | | 0.23 | | -0.17 | | -0.14 | | 0.13 | | -0.24 | | -0.13 | | -0.31 | |
| MD |  |  |  |  | |  | |  | |  | |  | |  | |  | |  | | ***** | | -0.54 | | -0.44 | | 0.23 | | **-0.61** | | 0.07 | | -0.05 | |
| DE |  |  |  |  | |  | |  | |  | |  | |  | |  | |  | |  | | ***** | | 0.02 | | 0.38 | | 0.50 | | 0.28 | | 0.07 | |
| HE |  |  |  |  | |  | |  | |  | |  | |  | |  | |  | |  | |  | | ***** | | 0.22 | | **0.68** | | 0.37 | | 0.26 | |
| forest |  |  |  |  | |  | |  | |  | |  | |  | |  | |  | |  | |  | |  | | ***** | | 0.37 | | **0.66** | | 0.11 | |
| elev |  |  |  |  | |  | |  | |  | |  | |  | |  | |  | |  | |  | |  | |  | | ***** | | 0.40 | | 0.24 | |
| slope |  |  |  |  | |  | |  | |  | |  | |  | |  | |  | |  | |  | |  | |  | |  | | ***** | | 0.51 | |
| rugged |  |  |  |  | |  | |  | |  | |  | |  | |  | |  | |  | |  | |  | |  | |  | |  | | ***** | |

**Table 13.** Spearman correlation coefficients between the predictor variables at transect-scale (1 km^2)^. Those with very high correlations (|r_s_| ≥ 0.70) were not used together in the same model. Number of sites (1-km transect) surveyed = 3,571 of 1 km^2^

|  | sambar | bovidae | allprey | domestic | road | village | agri | flat1km | flat3km | stream | slope | rugged | elev | MD | HE | DD | DE |
| --- | --- | --- | --- | --- | --- | --- | --- | --- | --- | --- | --- | --- | --- | --- | --- | --- | --- |
| sambar | ***** | **0.87** | **0.96** | -0.33 | 0.29 | **0.94** | -0.28 | 0.06 | -0.03 | -0.10 | 0.03 | -0.03 | 0.23 | -0.10 | 0.19 | 0.16 | 0.14 |
| bovidae |  | ***** | **0.99** | -0.31 | 0.28 | **0.96** | -0.26 | 0.01 | -0.08 | -0.05 | 0.06 | -0.02 | 0.37 | -0.19 | 0.29 | 0.14 | 0.19 |
| allprey |  |  | ***** | -0.31 | 0.27 | **0.98** | -0.25 | 0.05 | -0.03 | -0.07 | 0.01 | -0.07 | 0.32 | -0.17 | 0.26 | 0.17 | 0.17 |
| domestic |  |  |  | ***** | -0.08 | -0.31 | 0.16 | 0.01 | 0.07 | 0.01 | -0.09 | -0.08 | -0.01 | 0.00 | -0.07 | -0.05 | -0.08 |
| road |  |  |  |  | ***** | 0.27 | -0.16 | -0.04 | -0.13 | -0.03 | 0.12 | 0.12 | 0.05 | -0.04 | 0.13 | 0.01 | 0.08 |
| village |  |  |  |  |  | ***** | -0.26 | 0.08 | -0.01 | -0.12 | 0.02 | -0.05 | 0.18 | -0.09 | 0.19 | 0.19 | 0.13 |
| agri |  |  |  |  |  |  | ***** | 0.13 | 0.20 | -0.05 | -0.08 | -0.04 | -0.11 | -0.10 | -0.07 | -0.06 | -0.12 |
| flat1km |  |  |  |  |  |  |  | ***** | **0.76** | -0.50 | -0.50 | -0.42 | -0.38 | 0.23 | -0.12 | 0.15 | -0.19 |
| flat3km |  |  |  |  |  |  |  |  | ***** | -0.37 | **-0.66** | -0.56 | -0.36 | 0.22 | -0.17 | 0.16 | -0.16 |
| stream |  |  |  |  |  |  |  |  |  | ***** | 0.01 | 0.01 | 0.33 | -0.18 | 0.01 | -0.06 | 0.23 |
| slope |  |  |  |  |  |  |  |  |  |  | ***** | **0.85** | 0.26 | -0.04 | 0.20 | -0.15 | 0.11 |
| rugged |  |  |  |  |  |  |  |  |  |  |  | ***** | 0.16 | -0.04 | 0.13 | -0.16 | 0.09 |
| elev |  |  |  |  |  |  |  |  |  |  |  |  | ***** | -0.58 | 0.53 | -0.13 | 0.40 |
| MD |  |  |  |  |  |  |  |  |  |  |  |  |  | ***** | -0.29 | -0.04 | -0.50 |
| HE |  |  |  |  |  |  |  |  |  |  |  |  |  |  | ***** | -0.06 | -0.10 |
| DD |  |  |  |  |  |  |  |  |  |  |  |  |  |  |  | ***** | -0.11 |
| DE |  |  |  |  |  |  |  |  |  |  |  |  |  |  |  |  | ***** |
